# Supplementary material for: MetaRibo-Seq measures translation in microbiomes
Source: Nat Commun. 2020 Jun 29;11:3268. doi: 10.1038/s41467-020-17081-z (PMC7324362; doi:10.1038/s41467-020-17081-z)
Supplement: Supplementary file 10 — Supplementary Data 7 [file 41467_2020_17081_MOESM10_ESM.zip › File2/Confidence_VeryHigh_Taxonomy/203693_out.krona.html]

Javascript must be enabled to view this page.

members
magnitude
magnitudeUnassigned
count
unassigned
taxon
rank

203693\_out

4

superkingdom
4
2

phylum
1239
4

class
186801
4

order
4
186802

4
186803
family

39491

SRS015579\_contig\_number\_27050SRS016954\_contig\_number\_contig-100\_5.159701SRS022609\_contig\_number\_41920SRS062701\_contig\_number\_6781
4
species
